# Topical ozone application for severe acne with serological evidence of prior varicella-zoster infection after unsuccessful antibiotic and corticosteroid treatment: a case report

**DOI:** 10.1186/s13256-025-05386-w

**Published:** 2025-07-12

**Authors:** Taras Pyatkovskyy, Olena Pokryshko, Artur Markowski, Tetiana Chernyshova, Serhii Danylkov

**Affiliations:** 1 Department of Microbiology, Virology and Immunology, I. Horbachevsky Ternopil National Medical University of the Ministry of Health of Ukraine, Ternopil, Ukraine; 2 PCME Medical Center, Torun, Poland; 3 Holis-Med Medical Center, Torun, Poland; 4 Department of Family Medicine (General Practice), Bogomolets National Medical University, Kyiv, Ukraine

**Keywords:** Acne fulminans, Antimicrobial activity, Case report, Ozone, Varicella zoster

## Abstract

**Background:**

Acne fulminans is a rare and extreme form of inflammatory acne, which is characterized by the sudden onset of painful, ulcerative nodules and systemic symptoms such as fever, malaise, arthralgia, leukocytosis, and potential scarring.

**Case presentation:**

We present the case of a 16-year-old white European (Polish) female patient with acne vulgaris that progressively worsened despite treatment with oral isotretinoin, estrogens, topical antibiotics, and corticosteroids. She subsequently developed systemic complications, including fever, malaise, severe musculoskeletal pain, and tachycardia, and was hospitalized, where she received corticosteroids, antibiotics, and isotretinoin; however, the inflammatory and purulent processes persisted. Following discharge, her condition further deteriorated, presenting as severe swelling, pustules, and inflammatory lesions. Upon consulting a different clinic, all previous medications were discontinued, and a treatment regimen involving topical ozone was initiated. This included washing with ozonated water (2.4 ppm) and applying ozonated olive oil (peroxide index 2300) twice daily. The therapy resulted in a rapid reduction of inflammation and purulent lesions, with significant improvement observed within days.

**Conclusion:**

This case report suggests a potential benefit of combining ozonated water and ozonated oil as a novel therapeutic approach for refractory acne fulminans.

## Key summary points

- This case report describes the use of topical ozone application in the management of severe acne with apparent clinical benefit.
- Conventional treatments, including antibiotics, corticosteroids, and oral isotretinoin, did not control the patient’s condition and were associated with systemic complications.
- Topical application of ozonated water and ozonated olive oil was followed by rapid improvement in inflammation and purulent symptoms.
- This case provides preliminary observational evidence supporting the potential of ozone application as an adjunctive therapy in managing refractory acne, particularly when associated with viral factors or resistance to standard treatments.

## Introduction

Acne vulgaris is a common persistent chronic skin inflammatory condition of the pilosebaceous follicles, which affects 9.4% of the global population. It is particularly prevalent among teenagers, with over 85% of individuals experiencing some degree of acne due to hormonal changes that stimulate sebaceous gland activity [1, 2]. While most cases of acne vulgaris are mild-to-moderate and can be managed with topical therapy, severe complications such as acne fulminans can occur. Acne fulminans is rare and the most dangerous complication of acne vulgaris, often treated with oral isotretinoin [3]. This severe form poses a significant therapeutic challenge owing to its inflammatory nature and the increased risk of scarring [1]. Due to its low incidence, there is limited consensus on optimal management, particularly in cases that do not respond to standard therapies such as isotretinoin and corticosteroids [4]. Refractory presentations remain a therapeutic challenge, prompting clinicians to consider novel or adjunctive treatment approaches [5]. Although traditional therapies such as corticosteroids, retinoids, and antibiotics remain widely used, the alternative strategies are under active research. One such emerging treatment option is the application of ozone, which has demonstrated promising antimicrobial [6] and anti-inflammatory properties [7]. In dermatology, it can be used in the form of gaseous ozone, aqueous ozone solutions, and ozonated oils [8].

Here, we present a clinical case that demonstrates the effectiveness of topical ozone applications in treating severe acne associated with high anti-varicella-zoster immunoglobulin (Ig)G titers where traditional treatments proved to be ineffective.

## Presentation of the case

**Fig. 1:**
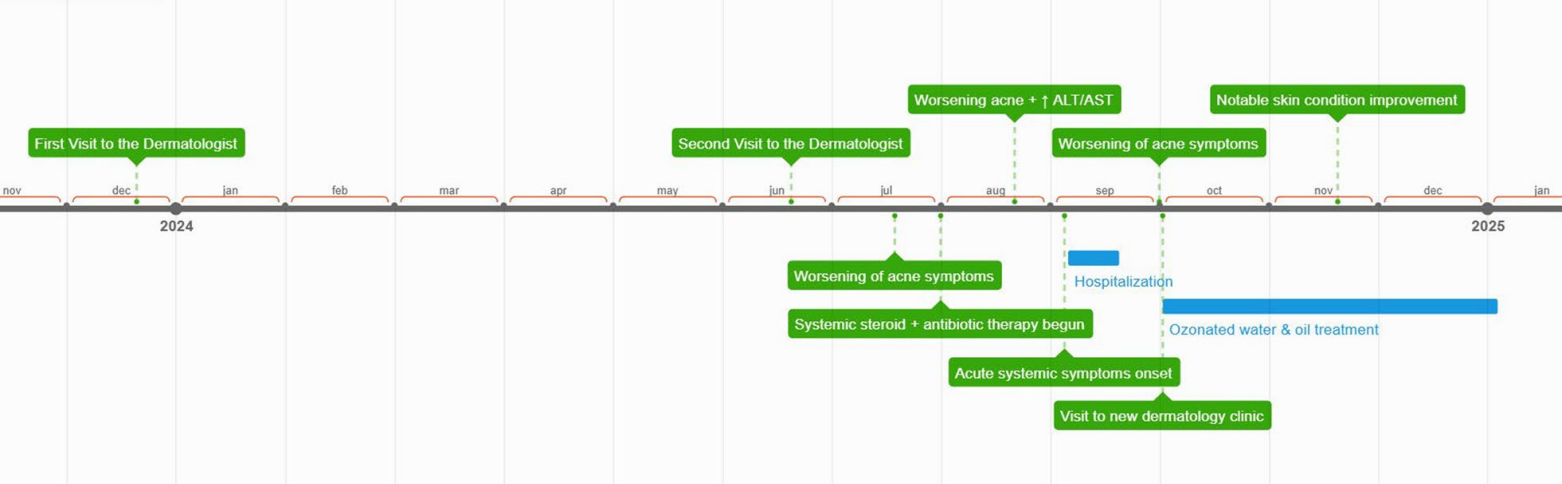
Timeline of clinical events, interventions, and outcomes

In December 2023, a 15-year-old white European (Polish) female from a family with an average income presented to a dermatologist with complaints of back acne and hair loss. There was no family history of acne. A detailed timeline summarizing the patient’s clinical course, treatments, and outcomes is presented in Fig. 1 to facilitate understanding of the sequence of events.

**Fig. 2:**
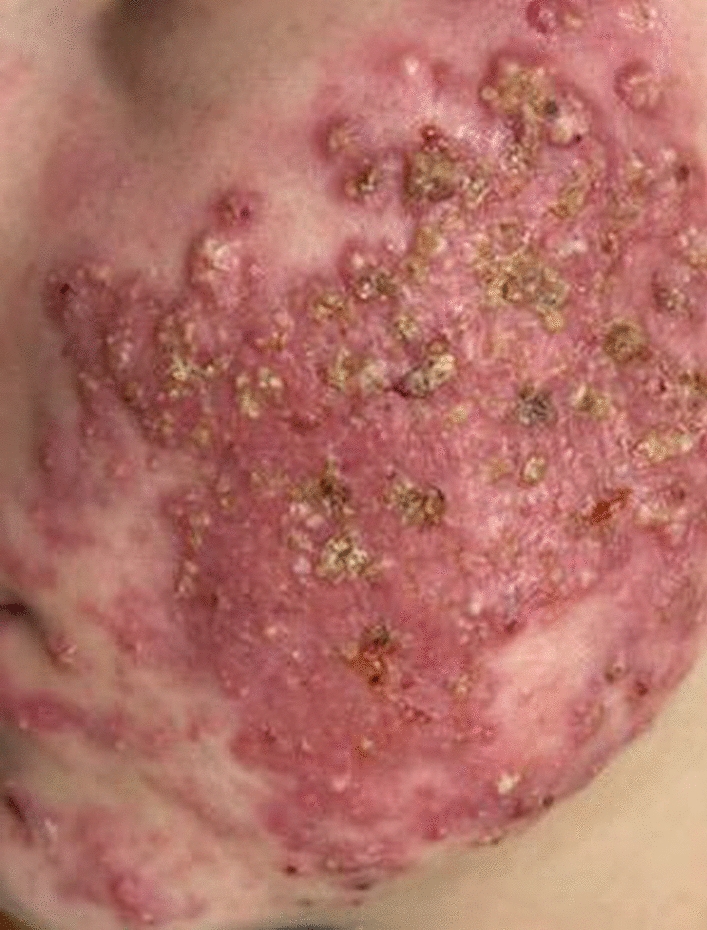
Clinical presentation of severe acne characterized by multiple inflammatory papules and pustules on the patient’s cheek prior to ozone application treatment. This condition affected the patient’s lips, chin, and forehead as well

She was prescribed gel formulations of adapalene/benzoyl peroxide and isotretinoin. However, owing to a delay in treatment initiation by the family, she only began taking the medications in May 2024. In June 2024, she returned to the clinic, reporting that the treatment had been ineffective. She was then prescribed oral isotretinoin (20 mg twice daily) and a combination of estrogens (ethinyl estradiol 0.02 mg and drospirenone 3 mg), along with over-the-counter topical medications. At 1 month later, she reported a worsening of her condition, characterized by dryness, inflammation, multiple cracks, and lymphatic leakage. The isotretinoin dose was subsequently halved. In August 2024, to improve her condition, she was prescribed prolonged steroids by injection (betamethasone dipropionate and betamethasone sodium phosphate), a 3-day course of the antibiotic azithromycin with weekly repetitions, isotretinoin 20 mg once daily, and clindamycin phosphate topical gel. After 3 weeks, the patient showed worsening inflammation on the facial skin and a marked increase in liver alanine transaminase levels (ALT): 140 IU/L (reference: 7–56 IU/L) and aspartate aminotransferase (AST): 123 IU/L (reference: 10–40 IU/L). Despite insignificant changes in the general blood test, these findings were interpreted as drug-induced hepatitis. As a result, betamethasone dipropionate and betamethasone sodium phosphate were reintroduced, and the isotretinoin dose was doubled. While laboratory abnormalities were consistent with hepatocellular injury, specific therapeutic adjustments addressing hepatic function were not implemented during this period. Following this adjustment, the formation and suppuration of pustules were observed. A total of 1 week later, the dose of isotretinoin was reduced to 20 mg daily, and azithromycin was added. Within 3 days, the patient developed spine and joint pain. In early September, isotretinoin was discontinued. The following night, the patient experienced a sudden worsening of her general condition, presenting with shortness of breath, chest pain radiating to the left arm, severe back and joint pain, general weakness, a temperature of 37.4 °C, and a heart rate of 150 beats per minute, leading to her admission to the hospital. Upon admission, laboratory tests revealed leukocytosis at 13.4 × 10^3^/μL (reference: 4.5–11.0 × 10^3^/μL), neutrophilia with neutrophils at 78.9% (reference: 40–70%), markedly elevated C-reactive protein (CRP) at 62.24 mg/L (reference: < 5.0 mg/L), and an elevated d-dimer level of 1509 ng/mL fibrinogen equivalent units (FEU; reference: < 500 ng/mL FEU). The hematological abnormalities indicated active systemic inflammation and suggested an increased risk of septic complications if not promptly addressed. Treatment included intravenous corticosteroids (prednisolone hemisuccinate), spiramycin, and isotretinoin. Nevertheless, the inflammatory and purulent manifestations persisted and showed only partial stabilization after 2 weeks. The patient was discharged from the hospital in late September 2024 with a prescription for corticosteroids (prednisone, for 4 weeks, starting at 20 mg daily and reduced by half each week), spiramycin (15 days), and isotretinoin. A total of 10 days later, the patient experienced increased swelling, inflammation, and purulent manifestations on the skin of the face and eyelids (Fig. 2).

**Fig. 3:**
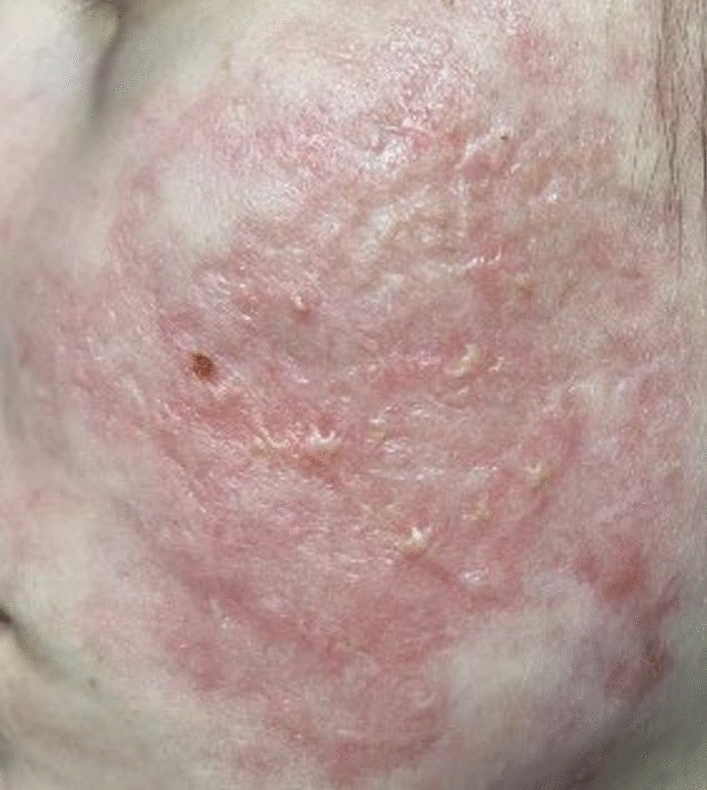
Significant improvement in the patient’s condition after 6 weeks of topical ozone application

Clinical examination revealed pronounced erythema with numerous inflammatory papules and pustules, many of which were covered with yellowish crusts suggestive of purulent content. Laboratory tests revealed a CRP level of 16.61 mg/L, mild neutropenia (44.8%), and mild lymphocytosis at 45.9% (reference: 20–40%), which are characteristic of viral process activation. Nevertheless, management at this point continued to focus primarily on acne and scarring prevention. In addition to oral isotretinoin, the patient was prescribed isotretinoin gel and scar-preventing ointment. The following day, the patient visited a different dermatology clinic. Tests revealed a high titer of antibodies to varicella-zoster virus (VZV) (IgG: 1044 mIU/ml, IgM: 0.4 mIU/ml), along with elevated CRP and d-dimer levels. Reference values for VZV IgG are typically considered positive above 100 mIU/mL [9]; thus, the patient’s value of 1044 mIU/mL indicates a strong seropositive status, suggestive of past exposure or reactivation. The prescribed treatment included discontinuation of all previous medications, anticoagulant therapy with low-molecular-weight heparins for 15 days, and initiation of topical ozone application. This consisted of two components. Ozonated water was generated on demand immediately before application using the Ozone Water Generator, model Y4 (ShenZhen BoRun Electronics Co., Ltd., Shenzhen, China), achieving an aqueous ozone concentration of 2.4 ppm. Wound washing with ozonated water was performed three times daily. In addition, commercially sourced ozonated olive oil (White Swan, Finland; peroxide index: 2300) was applied topically twice daily. The ozonated oil was prepared by the manufacturer using extra virgin olive oil exposed to continuous bubbling with gaseous ozone, resulting in a stable ozonide-rich formulation suitable for dermatological use. A reduction in inflammatory reactions and purulent processes was observed within the first few days of treatment. During the examination in November 2024, a significant reduction in the inflammatory reaction and a complete absence of purulent and necrotic elements were noted (Fig. 3).

Clinical examination revealed marked clearance of inflammatory lesions, with the absence of papules, pustules, and crusts. Residual erythema persisted, indicative of ongoing tissue remodeling and healing. No visible scarring was observed at that time, although the final cosmetic outcome remained to be determined. The treatment response was assessed through serial clinical evaluations and the patient’s self-reported outcomes, which were routinely collected during monthly follow-up consultations at the clinic and through periodic phone communication. To promote tissue regeneration, stimulate fibroblast proliferation, and minimize the formation of deforming scars, platelet-rich plasma and platelet-rich fibrin therapy was prescribed once a month after the signs of active inflammation had subsided. As of January 2025, the disease continues to regress. The patient is currently satisfied with the effectiveness of the new treatment and continues outpatient care aimed at the prevention of the scarring and improving skin trophism. She also continues to use ozonated water and ozonated oil as part of her personal skincare routine.

## Discussion

Acne vulgaris has a complex and multifaceted etiology that includes a mix of genetic susceptibility, hormonal fluctuations, lifestyle choices, and environmental influences. It usually starts with hyperkeratinized sebum obstructing hair follicles, followed by inflammation brought on by bacterial colonization [1]. First-line therapy often consists of topical medications such as azelaic acid, benzoyl peroxide, salicylic acid, retinoids, and antibiotics. Oral medications including hormones (oral contraceptives), antibiotics, and retinoids may be administered for the management of moderate and severe cases of acne [10]. Among the alternative methods of acne management were reported treatments with topical probiotics, plant derivatives, and protein derivatives [11, 12].

Our patient initially presented with severe inflamed acne resistant to conventional treatments, including topical and oral retinoids, corticosteroids, and antibiotics. Despite adherence to these regimens, her condition worsened, leading to significant inflammation, fever, chest pain, and severe back and joint pain. This condition, characterized by such systemic symptoms, is known as acne fulminans [3, 13].

Following consultation at a different dermatology clinic, serological testing revealed a high titer of anti-VZV IgG antibodies. While this finding indicates previous exposure or possible reactivation, no virological testing was performed to confirm active infection, and the patient did not receive antiviral therapy. Therefore, a direct relationship between VZV and the patient’s severe acne could not be established. However, it is worth noting that viral infections, including those caused by herpes viruses, may exert immunomodulatory effects that could influence the severity or course of inflammatory skin conditions [14, 15].

Ozone application, in the form of an electrolytic ozone aqueous solution and ozonated olive oil, has demonstrated antimicrobial, anti-inflammatory, and wound-healing properties. The therapeutic effects of ozone in dermatology are largely attributed to its potent oxidative properties [16, 17]. Ozone rapidly reacts with biological membranes, leading to lipid peroxidation, protein oxidation, and the inactivation of microbial enzymes. This oxidative damage compromises cell membrane integrity and disrupts microbial metabolic functions, resulting in broad-spectrum antimicrobial activity against bacteria, fungi, and viruses [18, 19]. The combined application of ozonated water and oil is guided by ozone’s half-life, which is much shorter in aqueous solutions [20, 21]. Conversely, ozonated oil releases ozone slowly over an extended period, thereby prolonging the therapeutic effect [22]. Furthermore, the gel-like form of ozonated oil helps soften hardened comedones and restore the functionality of the skin’s sebaceous glands. In our patient’s case, significant improvement was observed shortly after initiating this treatment. Our observations align with those of Ugazio *et al*., who described the use of ozonated oils in the treatment of various skin diseases [23]. The research conducted by Silva *et al*. demonstrated the antimicrobial and antibiofilm potential of ozonated vegetable oils against methicillin-resistant *Staphylococcus aureus* (MRSA) strains isolated from diabetic foot ulcers [24]. Similarly, Song *et al*. showed potent antistaphylococcal effects of ozonated camellia oil, both *in vitro* and in resolving MRSA-related skin infections following topical application [8]. In addition, Jian *et al*. reported the successful use of topical ozonated water and oil application in patients with herpes zoster, showing faster clinical improvement compared with conventional treatment with oral valacyclovir and topical mupirocin, and without notable side effects [25]. Furthermore, Ouf *et al*. investigated the antifungal efficacy of ozonated oil against common dermatophyte species, revealing that ozonated oils were more effective than gaseous ozone in treating superficial fungal infections of the skin, nails, and hair [26]. Ozonated water, produced by dissolving gaseous ozone in water [27] or through water electrolysis [17], has also been successfully used for antibacterial treatment and wound healing. For instance, Hu *et al*. reported its successful use in flushing diabetic foot ulcers [28]. The patient did not report any side effects of the proposed treatment, which is consistent with the findings of Puxeddu *et al*., who observed no cytotoxic effects of ozonated olive and sunflower oils on keratinocytes and epithelial cells, despite demonstrating a potent antimicrobial effect on specific microorganisms [29]. In a clinical trial, Lu *et al*. assessed the efficacy of commercial ozonated oil and laboratory-prepared ozonated water in treating tinea pedis. Their findings showed that there were no adverse effects and that this combination was successful in treating tinea pedis, as verified by mycological analysis [30]. It must be acknowledged that the patient’s clinical improvement could have been partially influenced by the discontinuation of systemic isotretinoin and corticosteroids, which have known potential to exacerbate inflammatory responses in rare cases [5]. In addition, spontaneous remission and a placebo effect, although less likely given the severity and progression of the condition, cannot be entirely excluded as a contributing factor. However, the observed improvement correlated temporally with the initiation of topical ozone therapy, and the known antimicrobial and anti-inflammatory properties of ozonated water and oil provide a plausible mechanism supporting its adjunctive therapeutic role in this case.

## Limitations

This report describes a single clinical case without a control group, and causality between the interventions and clinical improvement cannot be definitively established. The absence of virological confirmation of active varicella-zoster infection represents an additional limitation. Larger, controlled studies are needed to validate the clinical efficacy of ozonated water and oil in similar dermatological conditions.

## Conclusion

When first-line treatments for severe acne, such as antibiotics and corticosteroids, prove unsuccessful, alternative management strategies should be explored. One such approach may include topical ozone application, utilizing an electrolytic ozone aqueous solution and ozonated olive oil. This case report illustrates the challenges of managing acne fulminans, a severe form of acne that was refractory to conventional treatments. In this patient, the application of ozonated water and ozonated oil as part of the treatment protocol was associated with clinical improvement, including a reduction in inflammation and purulent processes. The observed effects may be related to the well-documented antimicrobial, antiviral, and anti-inflammatory properties of ozone. The combined use of ozonated water and oil potentially provided both immediate and prolonged therapeutic effects, through direct action and slow release of active ozone over time.

## Data Availability

The data supporting the findings of this case report are included within the article. Additional patient-related data are not available due to ethical and confidentiality considerations.
